# Sex Differences in Salivary Oxytocin and Cortisol Concentration Changes during Cooking in a Small Group

**DOI:** 10.3390/bs8110101

**Published:** 2018-11-03

**Authors:** Teruko Yuhi, Kosuke Ise, Kei Iwashina, Naoya Terao, Satoshi Yoshioka, Keijiro Shomura, Toshikatsu Maehara, Akari Yazaki, Kana Koichi, Kazumi Furuhara, Stanislav M. Cherepanov, Maria Gerasimenko, Anna A. Shabalova, Kouhei Hosoki, Hikari Kodama, Hong Zhu, Chiharu Tsuji, Shigeru Yokoyama, Haruhiro Higashida

**Affiliations:** 1 Department of Basic Research on Social Recognition, Research Center for Child Mental Development, Kanazawa University, 13-1 Takara-Machi, Kanazawa 920-8640, Japan; kou.conan@gmail.com (K.I.); kei.i@stu.kanazawa-u.ac.jp (K.I.); teraomedical058@gmail.com (N.T.); apollokun23@gmail.com (S.Y.); shomuracheckmate@gmail.com (K.S.); dmugen624@gmail.com (T.M.); yazakitty1995@gmail.com (A.Y.); cartah3beans@gmail.com (K.K.); furururukz.999@gmail.com (K.F.); stas4476@mail.ru (S.M.C.); mgera_08@mail.ru (M.G.); ashabalova@me.com (A.A.S.); khosoki473@gmail.com (K.H.); hikari1223.xas@gmail.com (H.K.); julia5444482@hotmail.com (H.Z.); ctsuji@med.kanazawa-u.ac.jp (C.T.); shigeruy@med.kanazawa-u.ac.jp (S.Y.); haruhiro@med.kanazawa-u.ac.jp (H.H.); 2 Laboratory for Social Brain Studies, Research Institute of Molecular Medicine and Pathobiochemistry, and Department of Biochemistry, Krasnoyarsk State Medical University named after Prof. V. F. Voino-Yasenetsky, 660022 Krasnoyarsk, Russia

**Keywords:** oxytocin, cortisol, group activity, in-group member, cooking, stress

## Abstract

**Background:** Oxytocin (OT), a neuropeptide, has positive effects on social and emotional processes during group activities. Because cooking is an integrated process in the cognitive, physical, and socio-emotional areas, cooking in a group is reported to improve emotion and cognition. However, evidence for efficacy in group cooking has not been well established at the biological level. **Methods:** To address this shortcoming, we first measured salivary levels of OT and cortisol (CORT), a biomarker of psychological stress, before and after group cooking for approximately 1 h by people who know each other in healthy married or unmarried men and women. We then compared the initial OT and CORT concentrations with those during individual non-cooking activities in isolation. **Results:** Baseline OT concentrations before group and non-group sessions did not significantly differ and OT levels increased after both types of activity in men and women. In men, however, the percentage changes of OT levels in the first over the second saliva samples were significantly small during cooking compared with those in individual activities. In women, however, such a difference was not observed. In contrast, the mean salivary CORT concentrations after group cooking were significantly decreased from the baseline level in both sexes, though such decreases were not significant after individual activity sessions. The sex-specific differences were marital-status independent. **Conclusion:** These results indicate that OT and CORT concentrations after two activity sessions by a familiar group changed in opposite directions in a sex-specific manner. This suggests that, because cooking is experience-based, we need to consider the sex-specific features of group cooking if we apply it for intervention.

## 1. Introduction

The nonapeptide oxytocin (OT) promotes social affiliation and bond formation, and functions to reduce anxiety and dampen the stress response in mammalian species [1,2,3,4,5,6,7,8,9,10,11,12,13,14,15]. OT also plays a role in mediating the stress-buffering and anxiolytic effects of close social interactions [16,17]. Impairments in the OT system are found in patients with autism spectrum disorder (ASD), borderline personality disorder, and anxiety disorders [3,18,19,20,21,22,23]. Nasal application of OT in subjects with ASD with or without comorbid intellectual disability has been shown to improve social interactions [24,25,26,27,28,29,30,31].

One way to understand the OT system in the nervous system is to measure OT concentrations in the brain or cerebrospinal fluid (CSF). We have reported that OT is released into the brain during social stress in mice [32]. Furthermore, OT concentrations in the CSF or extracellular fluid correlated positively with OT concentrations in blood plasma after experimentally induced stress, though baseline concentrations in blood may be reflected less in those in CSF [33]. As invasive methods are hard to apply in living humans, an alternative more practical non-invasive method is to measure OT concentrations in blood or salivary samples [34,35,36,37,38,39,40,41,42,43,44].

Pleasant activities in a group have beneficial effects in humans [45]. Therefore, not only during stressful conditions, it is hypothesized that OT can be released during pleasant activities in groups, especially with familiar members. There are several interesting reports on singing in a group [46,47,48,49]. Contrary to the initial expectation from the reports on the effects of singing referenced above, salivary OT increases during solo singing but not in singing as part of a choir [46]. This suggests that, although group singing seems to be an enjoyable experience and one that facilitates bonding [49], it can also be rather stressful because of having to pay attention to and account for the voices of other singers. In another case, we recently examined the biological effects of Japanese taiko drumming in a group as part of an educational intervention for children who are emotionally disturbed because of maltreatment. Taiko drumming can apparently improve the social behavior of these children, as judged by caregivers and school teachers [41]. Our results showed that the children’s mean salivary OT concentrations increased to various degrees after the recital but not much rehearsals. Group musical instrumental playing and choir singing are also reported to improve cognitive function, communication, impaired behavior, and mood [40,41,50,51,52,53,54,55,56,57,58], although the effects are not always consistent [59]. Therefore, we need to re-ask the question how group activity is simply comfortable to people by monitoring biomarkers, from the view of stress produced by group activity.

Cooking is an essential activity for daily living and a familiar activity for most people. Home cooking tasks are performed by one individual or by family members for the benefit of the family. Cooking is an integrated process in the cognitive, physical, and socio-emotional areas; therefore, kitchen skills or behaviors are useful for diagnosing cognitive function in geriatric psychiatry patients [60,61]. Furthermore, cooking is used as an intervention tool in therapy and rehabilitation [62,63,64]. Several studies that conducted cooking activities using people with dementia report statistically significant improvements in cognition and depression [63,65], because cooking is experience-based and pleasant feelings, while cooking may be ambivalent, i.e., pleasant and/or stressful.

To our knowledge, the beneficial effects of group cooking have not been measured by monitoring OT concentrations. Therefore, we first arranged a group cooking activity in a very small group as a pilot study. The group consisted of male and female healthy volunteers who were students and laboratory workers at a medical university who got acquainted in the authors’ laboratory. The activities were carried out during educational sessions as part of the curriculum during a 3-year period (2015–2017). After we noticed that, in men, increases in OT concentrations after cooking were not high in trials during 2015 and 2016, we added one experimental item in 2017 to measure saliva cortisol (CORT) concentrations, a biomarker of psychological stress, in the same saliva samples [66]. This was done to understand to what extent cooking is differentially stressful between men and women. In general, cooking is likely to be a pleasant activity for women but not for men. In addition, because cooking skill may not be similar between married and single people, we analyzed changes in OT and CORT concentrations by classifying participants by marital status in both sexes. Finally, we measured these concentrations during individual (free, usually desk or bench work) activities in isolated conditions for comparison.

## 2. Materials and Methods

### 2.1. Participants

The study recruited 31 healthy volunteer participants with an age cut-off of 21–69 years. Of the 31 recruited, we selected 18 (9 men [27.6 ± 5.3 years old] and 9 women [33.9 ± 3.9 years old], Table 1), for further statistical analysis based on more than 10 measures of OT and 5 measures of CORT (participants were third-year undergraduate students at Kanazawa University School of Medical Sciences and graduate students from Osaka University United Graduate School for Mental Development at the Kanazawa Campus. Others were workers and teachers from the Kanazawa University Research Center for Child Mental Development who engaged in educational sessions.) Ethnically, 14 participants were Japanese, 3 were Russian, and 1 was Chinese. Six participants were married and 12 were single (Table 1). During the tests, all participants got acquainted in the one author’s laboratory and became friends while staying together for 8 weeks to 12 years, as language or culture barriers decreased.

### 2.2. Ethics Statement

The study was approved by the student curriculum committee of Kanazawa University Graduate School of Medical Sciences in 2014 as educational sessions. The study was carried out during November to December in 2015, 2016 and 2017. All procedures involving human participants were conducted in accordance with the ethical standards of the institutional research committee and with the 1964 Helsinki Declaration. The participants were told that they could choose not to supply their saliva on each occasion.

### 2.3. Assessment

The participant’s salivary OT or CORT levels were assessed during 27 group cooking tasks and 11 sessions of individual activities other than cooking, such as desk or bench work (Supplementary Figure S1). This was done over 3 years, approximately twice a week. Experiments were started around 13:00. Saliva was collected during the first 10 min. At 2 to 5 min after participants rinsed their mouths with water, when their mouths were filled with newly secreted saliva, they bit down on a sterile 15-mL polypropylene tube (Greiner Bio-one Co. Ltd., Tokyo, Japan) and secreted saliva directly into the tube by chewing for 2–4 min [40,41]. This method was less stressful than using a cotton swab. Then they participated in an approximately 60-min cooking session in one room or in a 60-min individual activity in places of their choosing. Each cooking session was held with 4–10 participants (Supplementary Figure S1). Saliva was collected a second time 10 min after the end of each session. 

### 2.4. Saliva Collection and Analysis

The saliva samples (approximately 0.5–1.0 mL) in polypropylene tubes were kept on ice. They were then immediately frozen and stored at −80°C, as described previously [41]. At the assay time, they were thawed and centrifuged twice at 4 °C at 1500× *g* for 15 min. The samples were divided into 1.5-mL microtubes, each containing 100 µL [67].

Salivary OT was measured using a 96-plate commercial OT-ELISA kit (Enzo Life Sciences, Farmingdale, NY, USA), as described previously [41]. Saliva was not extracted in the current experiment because some studies have reported conducting measurements without extraction [40,68], as validated by MacLean et al. [69,70]. 

Measurements were performed in duplicate. The optical density of the samples and standards was measured at wavelengths of 405 and 590 nm by a microplate reader (Bio-Rad, Richmond, CA, USA). Sample concentrations were calculated by MatLab-7 according to the relevant standard curve [40].

It has been reported that some saliva components interact with the labelled OT in the assay mixture [69,71]. To test this, we first measured samples spiked with OT (0–250 pg/mL). A very high level was observed with no addition of spiked OT, suggesting an interaction with nonspecific antibody-interacting substances. Even with interacting substances in the samples, the enzyme immunoassay monitored concentrations were proportional to the spiked OT concentrations (Supplementary Figure S2), suggesting that monitored values are useful for calculating the ratio between values. Therefore, the difference between OT concentrations before and after the sessions seems to be more reliable.

### 2.5. Salivary cortisol assay

The same samples used for OT measurement were also used to measure CORT. Determination of saliva CORT was performed using a cortisol enzyme immunoassay kit (Salimetrics, State College, PA, USA), as described by Kumazaki et al. [67]. Samples (25 µL) were treated according to the manufacturer’s instructions. Measurements were performed in duplicate. The optical density of the samples and standards was measured at a wavelength of 450 nm by a microplate reader (Bio-Rad). Sample concentrations were calculated by MatLab-7 according to the relevant standard curve [40].

### 2.6. Statistical Analysis

Two-tailed Student’s *t*-tests were used for single comparisons between two groups. One- or two-way analyses of variance (ANOVA) were used for data with two or three components, respectively. Post hoc comparisons were performed only when the main effect was statistically significant. The *p*-values of the multiple comparisons were adjusted using Bonferroni’s correction. All data from in vivo and in vitro studies are shown as means ± s.e.m. In all analyses, *p* < 0.05 was taken to indicate statistical significance. All the analyses were performed using STATA data analysis and statistical software (Stata Corp. LP, College Station, TX, USA).

## 3. Results

### 3.1. Salivary Oxytocin Concentrations

Table 1 shows the characteristics of the participants in the group cooking study. Nine men (eight Japanese and one Russian) and nine women (six Japanese, two Russians and one Chinese) who were university students, postgraduate students, laboratory technicians and professors from two universities took part in the study in each November and December from 2015 to 2017. 

**Figure 1:**
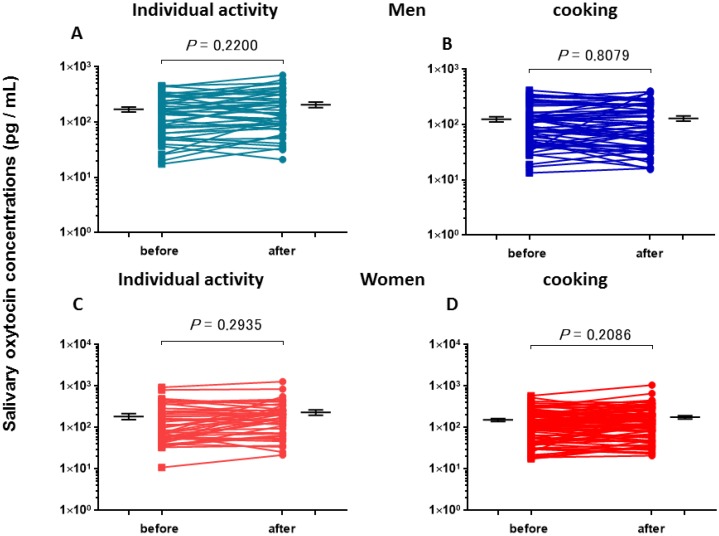
Oxytocin (OT) concentrations before and after a single group (cooking) or non-group (individual activity) session. OT levels are for the first (before) and second (after) salivary samples collected from nine men and nine women engaged in cooking in a group or in individual desk or bench work for approximately 1 h. *p*-values are for two-tailed Student’s *t*-tests: (**A**) individual activity by men, *p* = 0.2200, n = 46; (**B**) cooking by men, *p* = 0.8079, n = 55; (**C**) individual activity by women, *p* = 0.2935, n = 44; (**D**) cooking by women, *p* = 0.2086, n = 90.

The OT concentrations in the saliva collected before cooking in a group and the individual activity in isolation were determined and used as the baseline OT levels (Table 2). There were no significant differences among these values (one-way ANOVA, *F*_3,231_ = 1.93, *p* = 0.1250). The salivary OT concentrations measured before (first salivary samples) and after individual and cooking sessions (second salivary samples) were plotted in the male and female groups (Figure 1A–D). There were significant differences observed in the average OT concentrations among the four assessments of both sexes (one-way ANOVA, *F*_7,462_ = 3.15, *p* = 0.0029). Bonferroni *post hoc* tests revealed significant differences between the OT level after the individual activity in women (185 ± 31 pg/mL, n = 44) and the OT concentrations before and after cooking in men (121 ± 12, n = 55, 120 ± 14, n = 55, respectively; *p* < 0.016 for each value).

**Figure 2:**
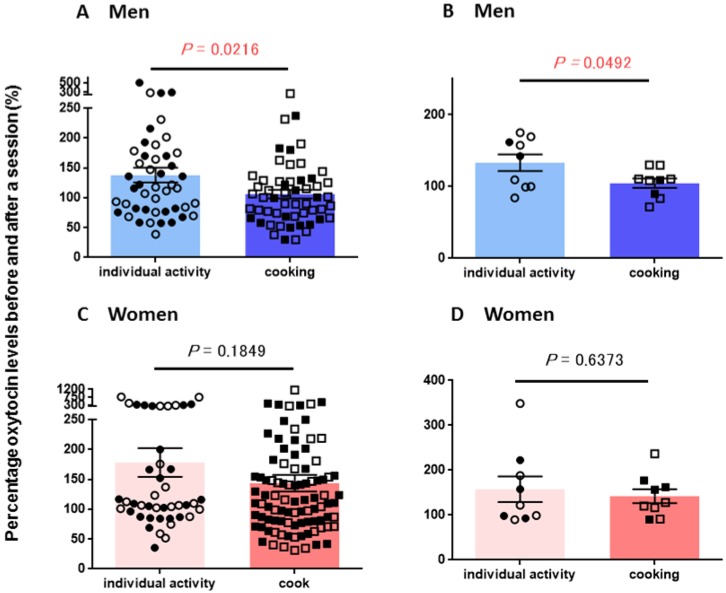
Percentage changes in oxytocin levels before and after a single session. Percentage of oxytocin levels for the first (before) saliva over the second (after) saliva samples after each session. Saliva was collected during individual (non-cooking) and group (cooking) activities in men (**A**) and women (**C**). The average percentage of oxytocin levels for the first (before) saliva over the second (after) saliva samples in individual male (**B**) and female (**D**) participants. *p*-values are for two-tailed Student’s *t*-tests: (**A**) *p* = 0.0216 between individual activity (n = 44) and cooking (n = 55) in men; (**B**) *p* = 0.0492, between individual activity (n = 9) and cooking (n = 9) in men; (**C**) *p* = 0.1849 between individual activity (n = 44) and cooking (n = 90) in women; (**D**) *p* = 0.6373 between individual activity (n = 9) and cooking (n = 9) in women. Filled and open symbols represent married and single participants, respectively.

The ratio of the salivary OT concentration changes was calculated by the values in the first (before) saliva over the second (after) saliva samples, as shown in Figure 1. Figure 2A shows percentage changes in 46 individual and 55 cooking sessions in men. OT concentration changes after cooking were significantly lower (two-tailed Student’s *t*-test, *p* = 0.0216) than those in the individual sessions. We further analyzed the average changes of the individual male participants. Figure 2B indicates that OT concentration increases after cooking were significantly lower (two-tailed Student’s *t*-test, *p* = 0.0182, n = 9 for each session).

The same analysis was performed for OT concentrations obtained in women. There were no significant differences in OT concentration changes between individual activity and cooking sessions as plotted in every measurement (Figure 2C, *p* = 0.1724, n = 43 and 90, respectively) nor in individual women (Figure 2D; n = 9, *p* = 0.2204).

Figure 2 shows marital status by indicating single (open symbols) or married (filled symbols) individuals. Judging from the bar graphs, the two symbols seem to be unevenly distributed and do not form any subpopulations. There were no differences between the average values in OT concentration changes before and after each activity in the married and single groups (Table 3). Two-way ANOVA showed no significant interaction between marital status and sessions on OT (*F*_3,227_ = 0.42, *p* = 0.7937). The results clearly indicate that OT concentration changes before and after cooking is correlated with male participants, but not to marital status. Therefore, in the last year (2017), we measured CORT levels in the saliva to test sex-relatedness in stress during cooking.

### 3.2. Salivary Cortisol Concentrations

**Figure 3:**
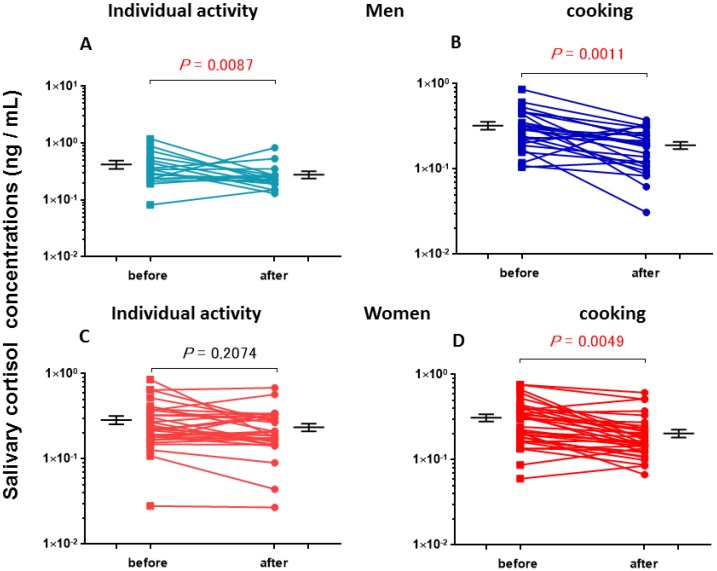
Cortisol concentrations before and after a single group (cooking) or non-group (individual activity) session. CORT levels are for the first (before) and second (after) saliva samples collected from five men and nine women engaged in cooking in group or in individual desk or bench work for approximately 1 h. *p*-values are for two-tailed Student’s *t*-tests: (**A**) individual activity by men, *p* = 0.0087, n = 17; (**B**) cooking by men, *p* = 0.0011, n = 26; (**C**) individual activity by women, *p* = 0.2074, n = 31; (**D**) cooking by women, *p* = 0.0049, n = 35.

Salivary CORT levels before and after cooking and non-cooking activities in male and female subjects were measured and are listed in Table 2. The baseline CORT concentrations before tasks were not significantly different in men and women (one-way ANOVA, *F*_3,105_ = 1.79, *p* = 0.1535). Furthermore, average CORT concentrations before and after individual activity sessions were not significantly changed in both men and women: Figure 3A,C; two-tailed Student’s *t*-test, *p* = 0.087 (n = 17) and *p* = 0.2074 (n = 31), respectively. However, percentage changes after cooking were significantly lower in male (*p* = 0.0011 (n = 26)) and female (*p* = 0.0049 (n = 35)) participants, compared with prior basal values (Figure 3B,D).

**Figure 4:**
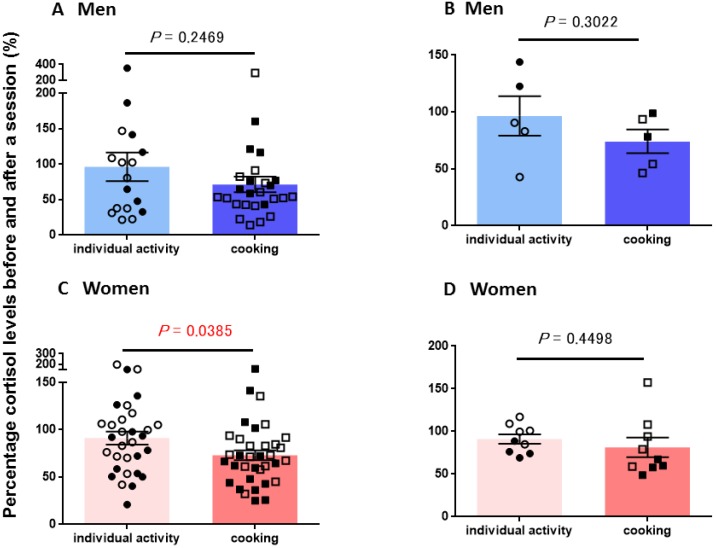
Changes in cortisol levels before and after a single session. Percentage of CORT levels for the first (before) saliva over the second (after) saliva samples after each session. Saliva was collected at individual (non-cooking) and group (cooking) activities in men (**A**,**B**) and women (**C**,**D**). Average percentage of cortisol levels for the first (before) saliva over the second (after) saliva samples in individual participants. *p*-values are for two-tailed Student’s *t*-tests: (**A**) *p* = 0.2469 between individual activity (n = 17) and cooking (n = 26) in men; (**B**) *p* = 0.3022 between individual activity and cooking in 5 men; (**C**) *p* = 0.0385 between individual activity (n = 31) and cooking (n = 35) in women; (**D**) *p* = 0.4498 between individual activity and cooking in 9 women. Filled and open symbols represent married and single participants, respectively.

The percentage of the salivary CORT concentration changes was calculated by the values in the first (before) saliva over the second (after) saliva samples, as shown in Figure 4. There were no significant differences in CORT concentration changes in men during both individual and cooking sessions as plotted in every measurement (Figure 4A, *p* = 0.2469, n = 17 and 26,) nor in individual subjects (Figure 4B; n = 5, *p* = 0.3022).

In women, average CORT levels in the second saliva compared with the first saliva samples were significantly lower after cooking than in individual activity sessions for every measurement (Figure 4C; *p* = 0.0385, n = 35 and 31). However, no significant difference was observed when compared for each individual (Figure 4D; *p* = 0.4498, n = 9). In addition, two-way ANOVA showed no significant interaction between marital status and sessions on CORT (*F*_3,108_ = 0.67, *p* = 0.5693).

## 4. Discussion

This study demonstrated that OT levels in men before and after cooking were nearly unchanged, while they increased during non-cooking tasks. Because OT levels during cooking increased in women, we assume that OT reflects a happy condition [72,73,74]; these findings suggest that cooking seems to be pleasant for women, but not so pleasant (actually rather stressful) for men. For women, cooking may not be extremely hard or stressful work, based on the lack of significant differences between their choosing work (individual activity) and cooking.

We observed sex differences in OT concentration changes before and after cooking. However, the differences were not dependent on marital status. Even though we inspected the data carefully, no clear differences between married and single lifestyles were observed, though it is easily assumed that cooking tasks in daily life are more familiar to married people. CORT concentrations after cooking were significantly lower in both men and women, suggesting that group cooking seems not to be stressful for either sex.

There is a circadian rhythm in CORT concentrations [75]; CORT concentrations are high in the morning, and gradually decrease in the afternoon. Therefore, it is not surprising that the CORT concentrations decreased after the 1-h period of each of the tasks. However, compared with the decrease after individual activities, the decrease after cooking was much greater in both men and women, suggesting that the decrease following cooking was additive of the amount from circadian rhythms and was therefore actually due to the cooking activity.

There is a report that compared music and cooking interventions in a randomized controlled trial of 48 patients with Alzheimer’s disease or mixed dementia [76]. Analyses revealed that both music and cooking interventions led to positive changes in the patients’ emotional state and decreased the severity of their behavioral disorders, as well as reduced caregiver distress. However, no benefit on the cognitive status of the patients was seen. This suggests that cooking interventions may have equivalent effects to music therapy. Unfortunately, they did not measure any biological markers in either intervention. Because cooking interventions are effective in the emotional domain, OT concentrations would have changed in their trials, likely similar to our results.

Group activities, usually with members who know each other or groups with fixed members, have beneficial effects from a therapeutic viewpoint. In the psychology and psychiatry fields, group therapy is used extensively within public mental health services [77,78]. It has been shown that cognitive therapy in groups improves the cognitive function of older people with dementia and depression [65,79,80,81]. Group musical instrumental playing and choir singing are reported to improve cognitive function, communication, impaired behavior, and mood [40,41,52,53,54,55,56,57,58,59,60], although the effects are not always consistent [59].

On the relationship between group activities and OT concentration changes, there are two relevant data. First, Kreutz [82] reported that a group of choir singers who singed together in a choir rehearsal for half an hour and on another occasion the same individuals sit talking in small groups together. The results showed that salivary OT increased after singing, with association of positive emotion increases and negative emotion decreases, while equivalent changes were not observed after talking. Second, it has been compared OT levels in amateur and professional singers during singing lessons [83]. Plasma OT concentrations increased both in amateurs and professionals after the lesson in the group.

Quantifying OT and CORT concentrations in saliva is a non-invasive and user-friendly sampling method [35,84,85] and is applicable to ASD subjects [41,67]. The precise relationship between OT concentrations in CSF remains to be elucidated, but it has been shown that situations induce OT release in the mouse brain more in the subordinate group than in the ordinate group according to social stress [86]. Increases in saliva OT concentration is observed following social stress [35].

Salivary CORT is recognized as a biomarker of stress response in a protective mechanism associated with acute and chronic stress [87,88] and CORT is released during stress [89]. CORT levels after control and cooking activities decreased in the current tests. The detected levels (~100 ng/mL) were identical to those reported for healthy adult males (88 ± 1.6 ng/mL; [90,91]). Kristenson et al. [92] reported that the correlation between CORT levels on consecutive 2- to 3-day periods was often in the order of 0.5. For reliable CORT levels, mean levels over 2–3 days are used. Unfortunately, we did not test in consecutive experiments; however, the CORT levels were the mean of several measurements in different days, which is more reliable than a single measurement. 

Using saliva samples in this pilot trial was a non-invasive method that made it possible to study a larger group of participants in the future to confirm the current results. However, cooking interventions are usually performed in small groups for practical reasons (e.g., a group using a kitchen in a nursing home), which makes it difficult to perform a large-scale cohort. 

This study did not address emotional changes after cooking. Such tests should be performed using a questionnaire survey. It is expected that the cooking was a pleasant experience because the products (mostly pickles, Supplementary Figure S1) from the cooking session were shared with the participants after the 3–10 days of pickling with salt or vinegar. In other words, all the participants knew they were to be rewarded with the products of their cooking. 

The investigation had some limitations. Though the salivary OT and CORT levels seem to have been affected, the menstrual cycle in female participants was not considered and sex hormone levels were not evaluated.

During the 3 years of this study, the participants were not divided into fixed groups and different numbers of participants joined each year. However, the participants were all acquainted and seven members of our laboratory started and ended the study together. It is not surprising that the groups were so fluid because many behavioral treatments in the community take place in open groups or a rolling format where group members join at different times and membership changes, as current study.

## Figures and Tables

**Table 1:** Demographic data.

	Men (Married)	Women (Married)
Number of participants	9 (2)	9 (4)
Each year		
2015	3 (1)	4 (3)
2016	4 (2)	6 (4)
2017	5 (2)	9 (4)
Age (years ± s.e.m. (range)) ^a^	27.6 ± 5.3 (21–69)	33.9 ± 3.9 (21–50)
Education (years ± s.e.m. (range)) ^b^	16.4 ± 0.96 (15–22)	16.1 ± 0.54 (14–20)
Ethnicity		
Japanese	8 (1)	6 (3)
Russian	1 (1)	2 (0)
Chinese	0	1 (1)

^a^*p* = 0.3466, two-tailed Student’s *t*-test; ^b^ *p* = 0.7657, two-tailed Student’s *t*-test.

**Table 2:** Baseline concentrations of oxytocin and cortisol.

		Men	Women
OT concentrations (pg/mL ± s.e.m.)			
Individual activities	before	170 ± 18 (46) ^a,b^	185 ± 31 (44) ^a,b^
	after	207 ± 24 (46) ^b,c^	234 ± 35 (44) ^b,c^
Cooking	before	125 ± 13 (55) ^a,b,c^	153 ± 12 (90) ^a,b^
	after	120 ± 14 (55) ^b,c^	178 ± 16 (90) ^b^
CORT concentrations (ng/dL ± s.e.m.)			
Individual activities	before	420 ± 69 (17) ^d,e,f^	286 ± 31 (31) ^d,e^
	after	278 ± 41 (17) ^e^	235 ± 24 (31) ^e,f^
Cooking	before	330 ± 34 (26) ^d,e,g^	312± 49 (35) ^d,e,h^
	after	190 ± 18 (26) ^e,f,g^	204 ± 21 (35) ^e,f,h^

^a^*p* = 0.1250, one-way ANOVA, among values labelled with ^a^; ^b^ *p* = 0.0029, one-way ANOVA, among values labelled with ^b^; ^c^ *p* < 0.01, Bonferroni *post hoc* test, for values labelled with ^c^; ^d^ *p* = 0.1564, one-way ANOVA, among values labelled with ^d^; ^e^ *p* = 0.0001, one-way ANOVA, among values labelled with ^e^; ^f^ *p* < 0.01, Bonferroni *post hoc* test, for values labelled with ^f^; ^g^ *p* = 0.0011, two-tailed Student’s *t*-test, between values labelled with ^g^; ^h^ *p* = 0.0049, two-tailed Student’s *t*-test, between values labelled with ^h^.

**Table 3:** Percentage of OT and CORT levels for the first (before) saliva over the second (after) saliva samples after each session in married and single participants.

	Men	Women
OT level changed (%±s.e.m.) ^a^		
Individual activities ^c^ (Married ^b^)	148 ± 22 (22)	153 ± 20 (23)
Individual activities ^c^ (Single ^b^)	128 ± 12 (24)	205 ± 45 (21)
Cooking ^c^ (Married ^b^)	102± 12 (22)	137 ± 12 (55)
Cooking ^c^ (Single ^b^)	108 ± 8 (38)	152 ± 32 (35)
CORT level changed (%±s.e.m.) ^d^		
Individual activities ^c^ (Married ^b^)	135 ± 42 (7)	82 ± 10 (14)
Individual activities ^c^ (Single ^b^)	69 ± 14 (10)	103 ± 9 (17)
Cooking ^c^ (Married ^b^)	87 ± 12 (9)	60 ± 8 (15)
Cooking ^c^ (Single ^b^)	63 ± 15 (17)	82 ± 6 (20)

^a^ A two-way ANOVA shows main effects of marital status of life style (^b^; *F*_3,235_ = 2.82, *p* = 0.0396) and two types (individual and cooking) of sessions (^c^; *F*_1,235_ = 4.29, *p* = 0.0396) without a significant interaction between marital status and sessions (*F*_3,235_ = 0.42, *p* = 0.7937) on percentage changes of OT concentrations before and after the sessions; ^d^ A two-way ANOVA shows main effects of marital status of life style (^b^; *F*_3,110_ = 3.76, *p* = 0.0131) and two types of sessions (^c^; *F*_1,110_ = 5.13, *p* = 0.0256) without a significant interaction between marital status and sessions (*F*_3,110_ = 0.67, *p* = 0.5693) on percentage changes of CORT concentrations before and after the sessions.

## Supplementary Materials

The following are available online at http://www.mdpi.com/2076-328X/8/11/101/s1, Figure S1: Cooking in a group performed by students from the medical school and laboratory workers. Figure S2: Salivary OT levels in adult samples after intestinal delivery of different concentrations of OT.
