# Supplementary figures and images for: Sex Differences in Salivary Oxytocin and Cortisol Concentration Changes during Cooking in a Small Group
Source: Behav Sci (Basel). 2018 Nov 3;8(11):101. doi: 10.3390/bs8110101 (PMC6262286; doi:10.3390/bs8110101)

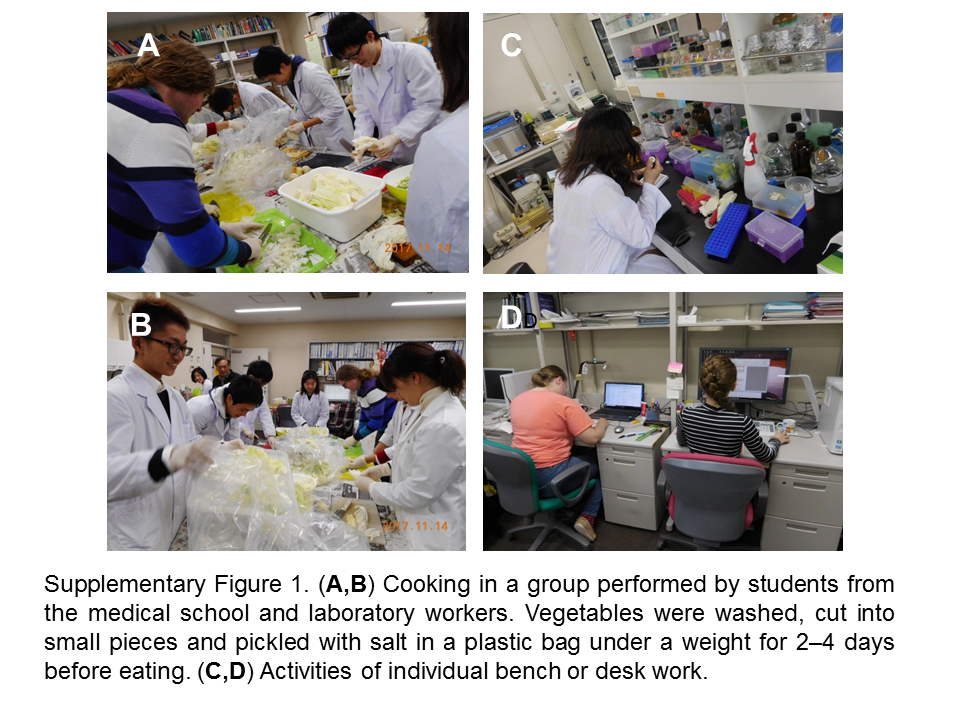

Supplement: Supplementary file 1 [file behavsci-08-00101-s001.zip › figures-supple/Supplemental Figure 1.TIF]

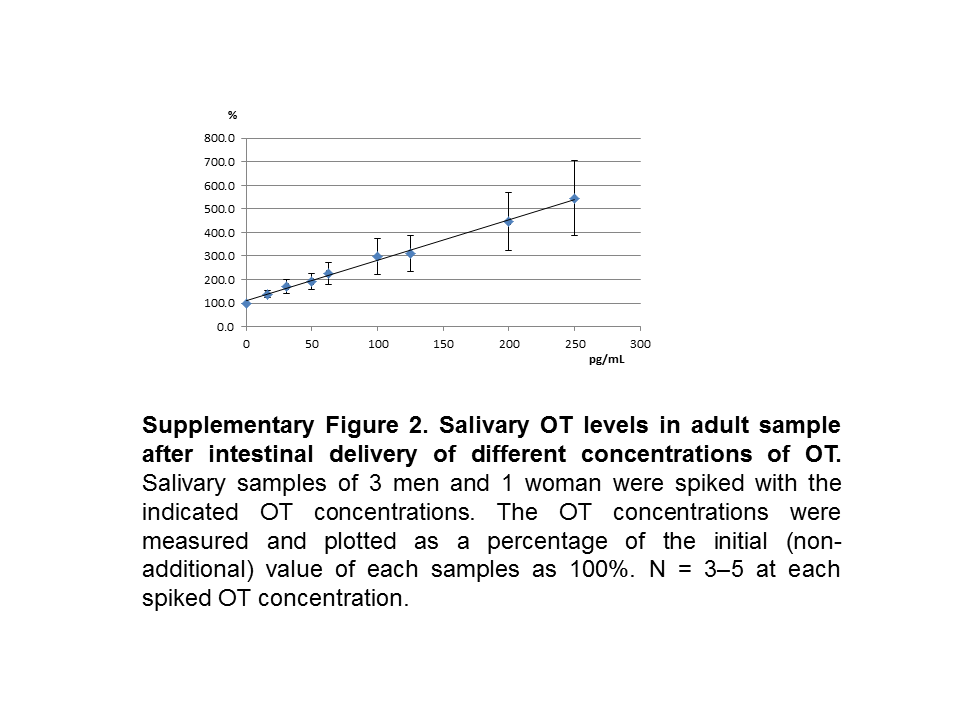

Supplement: Supplementary file 1 [file behavsci-08-00101-s001.zip › figures-supple/Supplemental Figure 2.TIF]
